# Meta-analysis: High-dose vs. low-dose metronidazole-containing therapies for *Helicobacter pylori* eradication treatment

**DOI:** 10.1371/journal.pone.0189888

**Published:** 2018-01-25

**Authors:** Yingjie Ji, Hong Lu

**Affiliations:** Division of Gastroenterology and Hepatology, Key Laboratory of Gastroenterology and Hepatology, Ministry of Health, Renji Hospital, School of Medicine, Shanghai Jiao Tong University, Shanghai, China; University Hospital Llandough, UNITED KINGDOM

## Abstract

**Objective:**

The purpose of this study was to evaluate the efficacy of high dose of metronidazole in the treatment of *Helicobacter pylori* (*H*. *pylori*) infection.

**Methods:**

Studies were identified from databases (Pubmed, Embase, Cochrane Library, ClinicalTrials.gov) searched from January 1990 to September 2017 using a battery of keywords. We included randomized controlled trials (RCTs) of H. pylori treatment comparing the high-dose and low-dose metronidazole-containing therapies (high-dose and low-dose therapies). Two reviewers independently selected studies, extracted relevant data and assessed study quality. A meta-analysis was performed by using Review Manager 5.3. Dichotomous data were pooled to obtain the relative risk (RR) of the eradication rate, with 95% confidence intervals (CIs).

**Results:**

Four randomized controlled trials, a total of 612 patients with a diagnosis of *H*. *pylori* infection were included. Overall the meta-analysis showed that both high-dose and low-dose therapies achieved similar efficacy of intention-to-treat (ITT) eradication rate 82% vs. 76%, RR 1.12 (95%CI: 0.96 to 1.30), P = 0.15, and adherence 94% vs. 94%, RR 1.00 (95%CI: 0.97 to 1.04), P = 0.81, but side effects were more likely in high-dose therapies [32% vs. 17%, RR 1.84 (95%CI: 1.17 to 2.88), P = 0.008]. In subgroup analysis, increasing the dose of metronidazole enhanced eradication rates in areas with high metronidazole resistance [74% vs 52%, RR 1.40 (95%CI: 1.08 to 1.82), P = 0.01] and in individuals with metronidazole-resistant strains [71% vs. 46%, RR 1.50 (95%CI: 1.02 to 2.19), P = 0.04].

**Conclusions:**

Both high-dose and low-dose therapies can achieve similar eradication rates and adherence and generally low-dose therapies cause fewer side effects. In populations with high metronidazole resistance, high dose of metronidazole can increase the eradication rates of *H*. *pylori* infection.

## Introduction

Therapy with either proton pump inhibitors (PPIs) or colloidal bismuth subcitrate (CBS) plus two antibiotics (metronidazole, tetracycline, amoxicillin, clarithromycin, levofloxacin or rifabutin) have been used as effective and economical therapeutic regimes for curing *Helicobacter pylori* (*H*. *pylori*) infections [1, 2]. However, according to the recent Maastricht V/Florence Consensus Report guidelines [3], increasing *H*. *pylori* resistance to antibiotics [4–9], especially to clarithromycin, levofloxacin and metronidazole, has undermined the efficacy of triple therapies containing these drugs. Resistance varies from country to country; for most developed countries [5, 10], clarithromycin resistance has reached high level (15–40%), while the metronidazole resistance remains lower (<40%). For most developing countries, metronidazole resistance can reach 60% or more [6, 11]. For individuals who have taken clarithromycin and/or metronidazole before are also at high risk of antibiotic resistance regardless of their population expectations [3].

To improve the eradication rate for *H*. *pylori*, several studies have been conducted to examine alternative strategies to increase efficacy, for example, by extending the treatment duration to 14 days [12], using sequential therapy instead of continuous therapy [13], using high dose or new generation of PPIs [14, 15], using tailored therapy instead of empiric chosen treatment [16], and using bismuth-containing quadruple therapies [17, 18]. However, there is no meta-analysis reporting whether increasing the dose of metronidazole can enhance the eradication rate of *H*. *pylori*. Currently, there are few publications of studies having assessed the role of high dose of metronidazole in *H*. *pylori* treatment, as well as the side effects and their conclusion are inconsistent [19–22]. Therefore, we conducted a meta-analysis to compare high-dose metronidazole-containing therapies (high-dose therapies) with low-dose metronidazole-containing therapies (low-dose therapies) not only regarding therapeutic effectiveness, but also on the adherence and side effects during the treatments.

## Methods

### Literature search

A systematic review of the evidence was performed in September 2017, including the period from January 1990 to September 2017. The electronic databases of PubMed, Embase and Cochrane Library were searched through the combination of a series of logic keywords and text words related to Helicobacter pylori, metronidazole, therapy, and randomized controlled trails (RCTs) (S2 File). The references of the most recent guidelines were identified manually [3]. Besides, we’ve also searched the following website manually to retrieve unpublished and ongoing studies: ClinicalTrails.gov (http://www.clinicaltrails.gov/).

### Study selection and eligibility criteria

After combining search results from different databases and removing duplicates by using EndNote reference manager, two investigators (Yingjie Ji and Hong Lu) independently reviewed all the retrieved abstracts and full texts to remove ineligible studies. If any disagreement was raised, it was worked out by consultation and discussion with another researcher until the difference was resolved. Inclusion criteria for papers in the meta-analysis were: (1) papers or abstracts had to report the results of comparative, randomized trials; (2) studies had to include at least two branches of treatment comparing a low dose of metronidazole with a higher dose in similar therapies; (3) therapies should include the combination of a PPI or CBS, metronidazole and either tetracycline or amoxicillin lasting for at least 7 days; (4) *H*. *pylori* infection had to be determined by biopsy and/or urea breath test (UBT) prior to treatment; (5) eradication had to be evaluated by biopsy and/or UBT at least 4 weeks after the end of treatment [23]; (6) studies had to report the eradication outcome and side effects of patients. For each selected publication, the data extracted were: publication year, area, study design, patient characteristics (treatment experience), number of patients in each treatment arm, drug regimen, duration of treatment, tests used to confirm *H*. *pylori* infection and eradication of infection, number of patients in whom *H*. *pylori* infection was successfully eradicated [intent-to-treat (ITT) and per-protocol (PP)analyses], number of patients who discontinued therapy due to side effects, and number of patients with side effects as defined within each included trial.

### Quality assessment

Two researchers (Yingjie Ji and Hong Lu) evaluated the methodological quality of the included studies independently, using the modified Jadad score [24], a scale ranging from 0 to 7 according to the descriptions of randomization (0–2 points), concealment allocation (0–2 points), blinding method (0–2 points) and reporting of patient withdrawals (0–1 points), in which points are awarded if: the study is described as randomized (+1) and double blinded (+1) with allocation concealment (+1), of means of which is described appropriately in detail (+1 for each point mentioned above) and there is a description of withdrawals giving number and reason in both groups (+1). The scores range from 1 to 3 was considered to be low quality, while scores range from 4 to 7 was presumed to be high quality. Discrepancies in the interpretation were resolved by consensus.

### Outcome measures

The primary goal of this study was to compare the effectiveness of *H*. *pylori* eradication (ITT and PP eradication rates), adherence and the incidence of side effects of high-dose vs. low-dose therapies. Subgroup analyses were performed to evaluate: (1) the efficacy of the therapies in areas with different metronidazole resistance; (2) the efficacy of the therapies for metronidazole-resistant and sensitive strains; (3) the side effects and adherence of the therapies on the eradication rates.

### Statistical analyses

The results of the studies were analyzed by using RevMan software version 5.3 (The Nordic Cochrane Centre, The Cochrane Collaboration, Copenhagen, 2014). To evaluate heterogeneity between pooled studies, we used both the Chi^2^ (χ2) test [p-value (P) less than 0.10 indicates significant heterogeneity] and inconsistency index (I^2^) statistic (a value of less than 40% represents low heterogeneity and a value of 75% or more indicates high heterogeneity) [25]. Statistical heterogeneity was measured by sensitivity analyses to illustrate if any clinical heterogeneity was responsible for such statistical difference. We conducted not only an ITT analysis, but also a PP analysis to assess clinical outcomes. We summarized dichotomous outcome measures as the relative risk (RR) along with 95% confidence intervals (95%CI) by RevMan 5.3. The outcomes were pooled using random effects model. All P<0.05 were considered statistically significant.

## Results

### Study characteristics and methodologies

**Fig 1:**
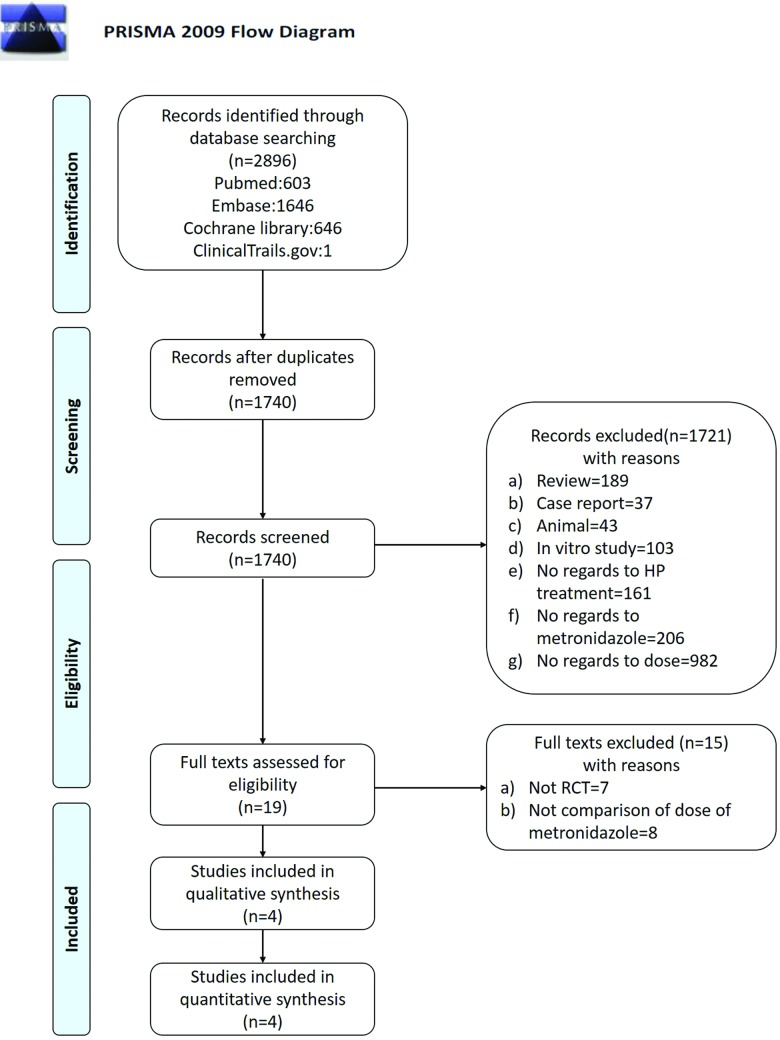
Flow chart of literature review.

The search of electronic databases and other sources in September 2017 resulted in 2896 studies. After combining the results, removing duplicates and selections based on the title and abstract, 19 full-text studies remained. Fifteen studies were excluded after reviewing the full text. Seven were excluded because they were not RCTs [26–32], eight were excluded because they were not comparison of high-dose vs. low-dose therapies [33–40]. After reviewing the potentially useful abstracts for the analysis, four studies, including 612 patients, with no publication bias (Egger’s test, P = 0.563, Begg’s test, P = 0.734), finally fulfilled the inclusion criteria and were included in this meta-analysis (Fig 1) [19–22]. The funnel plot of these trails is shown in S1 Fig. These trials were published between 1999 and 2006 (Tables 1 and 2).

**Table 1:** Characteristics of included studies.

Authors	Design	Area	Participants	Diagnostic Methods	U/NUD	Study Group (Male/Female)	Control Group (Male/Female)	Post eradication Test (Nw)
**H.Salman** **Roghan** **et al. [17]** **(1999)**	RCT	Iran	91 patients with endoscopically proven DU and positive 13C-UBT result.	1.RUT 2.Histology 3.Culture	91/0	n = 47(30/17) ages:39±12 years	n = 44(26/18) ages:42±9 years	1.RUT 2.Histology 3.Culture (4 weeks)
**Karna Dev** **Bardhan** **et al. [19]** **(2000)**	RCT	Europe	252 patients, who had an endoscopically proven DU and a positive 13C-UBT result.	1.13C-UBT 2.Culture	252/0	n = 125(77/50) ages:[18,49]:58,[50,64]:44,[65,+∞):25	n = 127(93/34) ages:[18,49]:52,[50,64]:47,[65,+∞):28	13C-UBT (4 and 8 weeks)
**E Fattahi** **et al. [18]** **(2004)**	RCT	Iran	41 patients with a history of endoscopy proven DU or dyspeptic symptoms and signs referable to the upper GI tract with no ulcer at endoscopy.	1.RUT 2.Microbiology 3.Histology	27/14	n = 18	n = 23	1.Histology 2.Microbiology 3.RUT(4 weeks)
**Takeshi** **Matsuhisa** **et al. [20]** **(2006)**	RCT	Japan	228 patients with HP infection in whom the first-line triple therapy with PPI+ AMX + CLA, failed to eradicate the infection, all of whom had undergone upper GI endoscopy before the start of the first-line eradication failure.	1.Histology 2.Culture 3.13C-UBT	174/54	n = 107(79/28) ages:51.2±10.7 years	n = 121(82/39) ages:55.7±12.1 years	13C-UBT (≥8 weeks)

RCT = randomized controlled trail, DU = duodenal ulcer, 13C-UBT = 13C urea breath test, GI = gastrointestinal, HP = helicobacter pylori, PPI = proton-pump inhibitor, AMX = amoxicillin, CLA = clarithromycin, RUT = rapid urease test, TET = tetracycline, U = ulcer,NUD = none-ulcer dyspepsia, Nw = time for testing after the end of treatment (in weeks).

**Table 2:** Characteristics of included studies.

Authors	Tailored Regiments	Empiric Controls	%Eradication Rate of Study Group (No. Patients)	%Eradication Rate of Control Group (No. Patients)	Side Effects in Study Group	Side Effects in Control Group
**H.Salman** **Roghan** **et al. [17]** **(1999)**	CBS 120mg tid +TET 500mg tid +MET 250mg tid, 14d	CBS 120mg tid + TET 500mg tid + MET 125mg tid,14d	ITT 70% (33/47) PP 77% (33/43)	ITT 46% (20/44) PP 50% (20/40)	Dry mouth 47%(20/43),Headache 9%(4/43),Skin rash 5%(2/43), Nausea 9%(4/43), Diarrhea 5% (2/43), Odynophagia 14%(6/43), Abdominal pain 21%(9/43)	Dry mouth 43%(17/40),Headache 5%(2/40),Skin rash 0%(0/40), Nausea 18%(7/40), Diarrhea 3% (1/40), Odynophagia 13%(5/40), Abdominal pain 5%(2/40)
**Karna Dev** **Bardhan** **et al. [19]** **(2000)**	OME 20mg bid + AMX 1000mg bid + MET 800mg bid,7d	OME 20mg bid + AMX 1000mg bid + MET 400mg bid,7d	ITT 83% (104/125) PP 85% (96/113)	ITT 76% (97/127) PP 81% (95/118)	Diarrhea/loose stool 35%(40/113), Taste perversion 9% (10/113),Liver lesion 7%(8/113),Headache 8% (9/113),Nausea 9%(10/113)	Diarrhea/loose stool 30%(35/118) Taste perversion 3% (3/118),Liver lesion 10%(12/118),Headache 6% (7/118),Nausea 3%(3/118)
**E Fattahi** **et al. [18]** **(2004)**	OME 20mg bid + AMX 1000mg bid + MET 500mg tid,14d	OME 20mg bid + AMX 1000mg bid + MET 250mg tid,14d	ITT 83% (15/18) PP 83% (15/18)	ITT 65% (15/23) PP 65% (15/23)	Epigastric pain or metallic taste 39%(7/18)	Epigastric pain or metallic taste 13%(3/23)
**Takeshi** **Matsuhisa** **et al. [20]** **(2006)**	PPI(OME 20mg bid, LAN 30mg bid, or RAB 10mg bid)+ AMX 750mg bid + MET 250mg tid,7d	PPI(OME 20mg bid, LAN 30mg bid, or RAB 10mg bid)+ AMX 750mg bid + MET 250mg bid,7d	ITT 87% (93/107) PP 77% (93/105)	ITT 88% (106/121) PP 91% (106/117)	Soft stool, diarrhea 24%(25/105), Abdominal pain 3%(4/105), Taste perversion 8%(8/105), Eruption 3%(3/105), Constipation 0%(0/105), Glossitis 1%(1/105), Total 30%(32/105)	Soft stool, diarrhea 8%(9/117), Abdominal pain 7%(8/117), Taste perversion 3%(3/117), Eruption 2%(2/117), Constipation 2%(2/117), Glossitis 0%(0/117), Total 18%(21/117)

MET = metronidazole, OME = omeprazole, LAN = Lansoprazole, RAB = rabeprazole, CBS = colloidal bismuth subcitrate, TET = tetracycline, ITT = intention-to-treat, PP = per protocol

Among them, 297 patients received high-dose therapies, whereas 315 received lower-dose therapies. In terms of areas, two studies were reported from Iran [19, 20], other two from Europe [21] and Japan [22]. In terms of durations, two studies were given 7 days [21, 22], while other two 14 days [19, 20]. In brief, one of them compared metronidazole 250 mg three times a day (tid) to metronidazole 125 mg tid [19], one metronidazole 800 mg twice a day (bid) to metronidazole 300 mg bid [21], one metronidazole 500 mg tid to metronidazole 250 mg tid [20], and one metronidazole 250 mg tid to metronidazole 250 mg bid [22]. One study used a combination of CBS, tetracycline and metronidazole [19], other three combined a PPI (omeprazole 20 mg bid, lansoprazole 30 mg bid, or rabeprazole 10 mg bid), clarithromycin and metronidazole [20–22]. Eradication was tested by UBT or endoscopy [rapid urease test (RUT), histology and culture] at least 4 weeks after the treatment. Two studies showed a significant improvement in eradication rates when using high-dose therapies [19, 20], meanwhile another two showed no differences in eradication rates [21, 22]. The methodological quality assessment (Table 3) showed that two studies belonged to low quality (score 3) [19, 21], and two belonged to high quality (score 4 or 5) [20, 22].

**Table 3:** Quality assessment of included studies.

Study	Randomized method	Concealment allocation	Blinding method	Reporting of participant withdraws	Total score
**Roghani [17]**	2	1	0	1	4
**Bardhan [19]**	2	2	0	1	5
**Fattahi E [18]**	1	1	0	1	3
**Matsuhisa [20]**	1	1	0	1	3

### Eradication rates of high vs. low-dose therapies

**Fig 2:**
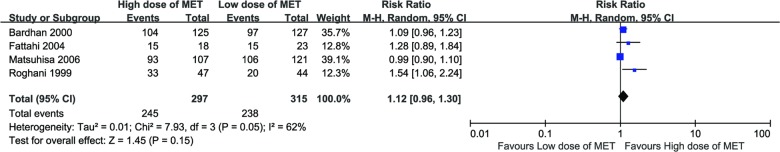
Forest plot of H. pylori eradication rate (intention-to-treat, ITT) with high-dose therapies compared to low-dose therapies.

Six hundred and twelve patients were included in the ITT analysis, eradication rates of high-dose therapies were 82% (245/297, 95%CI: 78%-87%) vs. 76% (238/315, 95%CI: 71%-80%) with the low-dose therapies (7% increase in eradication rates, 95%CI: 3%-11%). Pooling the ITT eradication rates of the four studies, the test for heterogeneity was positive (χ^2^ = 7.93, P = 0.05, I^2^ = 62%) and hence a random effects model was used. The RR for curing the infection with the high-dose therapies was 1.12 (95%CI: 0.96–1.30, P = 0.15). The Forrest plot for the ITT analysis is shown in Fig 2. The funnel plot showed no evidence of publication bias.

**Fig 3:**
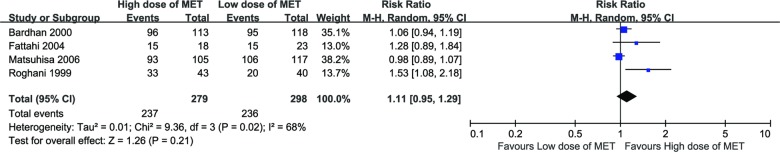
Forest plot of H. pylori eradication rate (per-protocol, PP) with high-dose therapies compared to low-dose therapies.

In the PP analysis, five hundred and seventy seven patients were involved. Eradication rates were 85% (237/279, 95%CI: 81%-89%) in the high-dose therapies vs. 79% (236/298, 95%CI: 75%-84%) in the low-dose therapies (6% increase in eradication rate, 95%CI: 2%-10%). The test for heterogeneity was positive (χ^2^ = 9.36, P = 0.02, I^2^ = 68%). A random effects model was used to analyze the PP eradication rates. The RR for eradicating the *H*. *pylori* infection with the high-dose therapies was 1.11 (95%CI: 0.95–1.29, P = 0.21), with no publication bias shown in the funnel plot. The Forrest plot for the PP analysis is shown in Fig 3.

### Sensitivity analyses

We performed a sensitivity analysis in which we excluded one study at a time. The sensitivity analyses did not change either the direction or the statistical significance of any of the RRs or the level of heterogeneity in any of the analyses, which was attributed to the insufficient sample size of all the included publications.

### Eradication rates in areas with high vs. low metronidazole resistance

**Fig 4:**
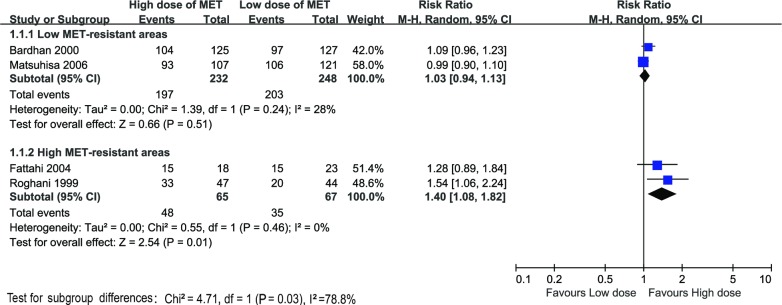
Forest plot of H. pylori eradication rate with high-dose therapies compared to low-dose therapies in the areas with high vs. low metronidazole resistance. A. Comparison of the high-dose therapies with low-dose therapies in low metronidazole-resistant areas. B. Comparison of the high-dose therapies with low-dose therapies in high metronidazole-resistant areas.

Two studies including 480 patients compared the eradication rates in areas with low metronidazole resistance (34.9% in Europe [8] and 5%-12% in Japan [41]) [21, 22].The ITT eradication rates were similar in high-dose therapies 85% (197/232, 95%CI: 80%-90%) and in low-dose therapies 82% (203/248, 95%CI: 77%-87%). The RR was 1.03 (95%CI: 0.94–1.13, P = 0.51), with negative heterogeneity (χ^2^ = 1.39, P = 0.24, I^2^ = 28%). While in other two studies from high metronidazole-resistant area (61.6% in Iran [6]) [19, 20], eradication rates of the high-dose therapies were 74% (48/68, 95%CI: 63%-85%) vs. 52% (35/67, 95%CI: 40%-64%) of the low-dose therapies (22% increase in eradication rate, 95%CI: 11%-32%). The RR was 1.40 (95%CI: 1.08–1.82, P = 0.01), and the heterogeneity tests was negative (χ^2^ = 0.55, P = 0.46, I^2^ = 0%). The Forrest plot of the subgroup analysis of different eradication rates in different areas is shown in Fig 4.

### Eradication rates in metronidazole-resistant vs. susceptible strains

Two studies reported different eradication rates of metronidazole-resistant and metronidazole-susceptible strains [19, 21], testing by the agar dilution method and minimum inhibitory concentration 8mg/L was regarded as threshold [42]. The eradication rates for the metronidazole-resistant strains in the high-dose therapies were 71% (27/38, 95%CI: 57%-85%), which were superior to that of the low-dose therapies 46% (19/41, 95%CI: 31%-62%), (25% increase in eradication rates, 95%CI: 10%-39%). The RR was 1.50 (95%CI: 1.02–2.19, P = 0.04), with negative heterogeneity (χ^2^ = 0.11, P = 0.74, I^2^ = 0%).

**Fig 5:**
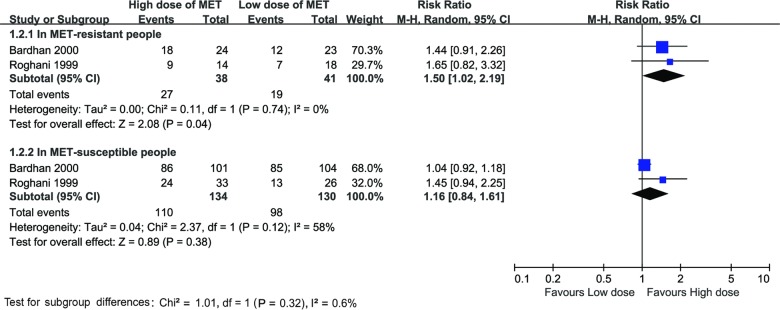
Forest plot of H. pylori eradication rate with high-dose therapies compared to low-dose therapies in the metronidazole-resistant vs. susceptible people. A. Comparison of the high-dose therapies with low-dose therapies in metronidazole-resistant people. B. Comparison of the high-dose therapies with low-dose therapies in metronidazole-susceptible people.

Another two hundred and sixty four patients proved to habour metronidazole-susceptible strains. The eradication rates for the metronidazole-susceptible stains in high–dose therapies were 82% (110/134, 95%CI: 76%-89%), which were similar to that of the low-dose therapies 75% (98/130, 95%CI: 68%-83%). The RR was 1.16 (95%CI: 0.84–1.61, P = 0.38), with the positive heterogeneity (χ^2^ = 2.37, P = 0.12, I^2^ = 58%). The Forrest plot of the subgroup analysis for this difference were shown in Fig 5.

### Adherence and side effects

**Fig 6:**
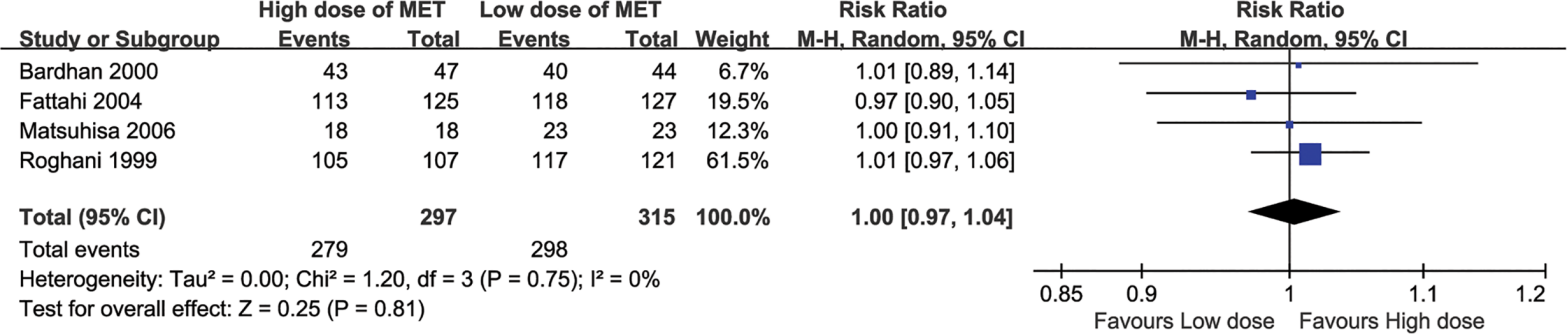
Forest plot of adherence difference between high-dose therapies and low-dose therapies.

Adherence was evaluated in all four included studies. Both therapies displayed a high adherence, with 94% (279/297, 95%CI: 92%-97%) for the high-dose therapies, and 94% (298/315, 95%CI: 92%-97%) for low-dose therapies. The RR was 1.00 (95%CI: 0.97–1.04, P = 0.81), no significant difference was observed in the meta-analysis, and the heterogeneity was negative among the studies (χ^2^ = 1.20, P = 0.75, I^2^ = 0%). The Forrest plot of the adherence of each group was shown in Fig 6.

**Fig 7:**
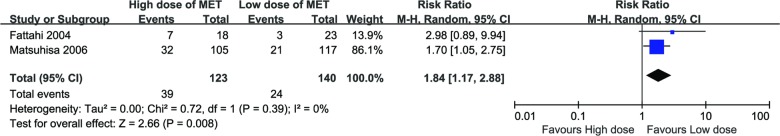
Forest plot of high-dose therapies vs. low dose-dose therapies in total side effects.

Only two studies described the total events of side effects [20, 22]; the high-dose therapies increased the risk of side effects (RR = 1.84, 95%CI: 1.17–2.88, P = 0.008). The overall side effects rate was 32% (39/123, 95%CI: 23%-40%) for high–dose therapies, and 17% (24/140, 95%CI: 11%-23%) for low-dose therapies (15% increase in eradication rate, 95%CI: 6%-23%). But there was no significant heterogeneity observed in the meta-analysis (χ^2^ = 0.72, P = 0.39, I^2^ = 0%). The Forrest plot of the total side effects was described in Fig 7.

The detailed information of each side effect is shown in S2 Fig. Dry mouth was the most common side effects for both high-dose (47%) and low-dose therapies (43%). Others like soft stool or diarrhea (27% vs. 19%), odynophagia (14% vs. 13%), abdominal pain or taste prevention (10% vs. 6%), nausea (9% vs. 6%) and headache (8% vs. 6%) tended to appear frequently during the treatment. Besides, side effects like abnormal liver function (i.e., serum Alanine transaminase [ALT] and/or Aspartate transaminase [AST] activity increased [43]) (7% vs. 6%), skin rash (3% vs. 1%), golssitis (1% vs. 0%) and constipation (0% vs. 2%) were rarely observed in patients receiving either high-dose or low-dose therapies.

## Discussion

### Summary of evidence

In the last 30 years the standard *H*. *pylori* regimen which includes a PPI and/or bismuth plus clarithromycin and amoxicillin or metronidazole was often regarded as the first choice of treatment for eradication of *H*. *pylori* [44, 45]. However, due to increasing primary resistance to these antibiotics, the eradication rates have generally declined to unacceptable levels [46, 47], especially for the dual clarithromycin and metronidazole-resistant strains. It has led some researchers to find new ways to enhance the eradication rates, for example, by increasing the dosage of metronidazole.

This meta-analysis included four prospective RCTs with 612 patients (297 in the high-dose therapies, and 315 in the low-dose therapies). The eradication rates (ITT, 82% vs. 76%, P = 0.15; PP, 85% vs. 79%, P = 0.21) and adherence (94% vs. 95%, P = 0.81) were not significantly different between these two groups. This finding suggest that regimens with both high dose and low dose of metronidazole might be considered equally effective. However, high dose of metronidazole resulted in more adverse events than that of low-dose groups (32% vs. 17%, P = 0.008). Subgroup analysis found that the eradication rates of these groups tend to be similar (ITT, 85% vs. 82%, P = 0.51) in areas like Europe or Japan [21, 22], where resistance to metronidazole was lower. In contrary, The ITT eradication rates of high-dose therapies was superior to that of low-dose therapies (74% vs. 52%, P = 0.01) in the areas like Iran, in which the prevalence of metronidazole-resistant strains is higher. Besides, the results of the present studies showed that the eradication rates of the high-dose therapies in Iran is 74%, lower than that in Europe and Japan. Thus, we propose that in the areas with high metronidazole resistance, higher dose of metronidazole would achieve higher eradication rates than that of low-dose ones, while in areas with low metronidazole resistance, low dose of metronidazole can also reach high eradication rates.

Antibiotic resistance is considered to be one of the main reasons for eradication failure [48, 49]. Thus, it is important to perform antimicrobial susceptibility test before treatment. Two of our included studies involving 343 patients conducted an antimicrobial susceptibility test [19, 21]. In the metronidazole-resistant subgroup (79 strains), we found that high-dose therapies reached a higher eradication rate than low-dose ones (71% vs. 46%, P = 0.04), with obvious statistical significance. But for the metronidazole-susceptible subgroup (264 strains), the eradication rates proved to be more similar between the two groups (82% vs. 75%, P = 0.38). The results shows that high dose of metronidazole can partially overcome the metronidazole resistance and reach a higher eradication rate, especially in areas with high metronidazole resistance or for individuals who had taken metronidazole before without antimicrobial susceptibility test.

Adherence is also one of the factors that determine the efficacy of eradication therapy [50]. Overall, no difference emerged among both groups (94% vs. 95%, P = 0.81) as good adherence (>94%) was observed in patients in all four involved studies. However, our meta-analysis found that high-dose therapies would cause higher incidence of side effects than low-dose therapies (total events, 32% vs. 17%, P = 0.008). Although the side effects appear more frequently in high-dose therapies, there was no difference in the adherence of both groups, which means that the patients using high dose of metronidazole can tolerate all the side effects mentioned above and can also complete the entire course of the treatment.

### Strengths and limitations

To diminish bias, the study selection, data extraction and evaluation of the study quality were performed by two reviewers separately. We comprehensively analyzed the efficacy of high-dose therapies. Sensitive analyses helped to make the outcomes of our meta-analysis reliable, and the subgroup analysis helped us to investigate the impact of high dose of metronidazole in population with different antibiotic resistance.

There were also several potential limitations of our meta-analysis. First, some well-designed studies were excluded because they were not published in English. Second, the sample size was small as only 4 RCTs were included in our meta-analysis. Although the studies were from different geographic locations that spanned Europe, Middle East and Asia, the results might have been more convincing if more RCTs had been analyzed. Third, the bias of the publications might have affected the validity of our conclusions, such as lack of double blinding. Fourth, individual studies included in our analysis differed in a few respects, such as inclusion and exclusion criteria, different PPIs, duration and combined antibiotics. Furthermore, largescale randomized controlled trials are warranted to compare therapeutic efficacy between high-dose and low-dose therapies.

## Conclusions

In summary, in some regions low dose metronidazole-containing therapies can achieve good eradication rates, with good adherence and fewer side effects. But in areas with high metronidazole resistance or for individuals who had taken metronidazole before without antimicrobial susceptibility test, increasing the dose of metronidazole can partially overcome the resistance and result in a higher eradication rates than use of low-dose therapies with the same adherence.

## Supporting information

S1 File Figure legends. (DOCX)

S2 File Appendix 1. Search strategies for Pubmed, EMBASE and The Cochrane Library database.
(DOCX)

S1 Fig Funnel plot assessing publication bias. (TIF)

S2 Fig Forest plot of high-dose therapies vs. low-dose therapies in particular adherence effects. (TIF)

S1 Table PRISMA checklist. (DOC)

## References

[1] De Boer WA, Tytgat GN. The best therapy for Helicobacter pylori infection: should efficacy or side-effect profile determine our choice? Scandinavian journal of gastroenterology. 1995;30(5):401–7. Epub 1995/05/01. . 7638563 10.3109/00365529509093298

[2] Suzuki H, Nishizawa T, Hibi T. Helicobacter pylori eradication therapy. Future microbiology. 2010;5(4):639–48. Epub 2010/04/01. doi: 10.2217/fmb.10.25 . 20353303 10.2217/fmb.10.25

[3] Malfertheiner P, Megraud F, O'Morain CA, Gisbert JP, Kuipers EJ, Axon AT, et al Management of Helicobacter pylori infection-the Maastricht V/Florence Consensus Report. Gut. 2017;66(1):6–30. Epub 2016/11/02. doi: 10.1136/gutjnl-2016-312288 . 27707777 10.1136/gutjnl-2016-312288

[4] Kobayashi I, Murakami K, Kato M, Kato S, Azuma T, Takahashi S, et al Changing antimicrobial susceptibility epidemiology of Helicobacter pylori strains in Japan between 2002 and 2005. Journal of clinical microbiology. 2007;45(12):4006–10. Epub 2007/10/19. doi: 10.1128/JCM.00740-07 . 17942652 10.1128/JCM.00740-07 PMC2168569

[5] Lee JW, Kim N, Kim JM, Nam RH, Chang H, Kim JY, et al Prevalence of primary and secondary antimicrobial resistance of Helicobacter pylori in Korea from 2003 through 2012. Helicobacter. 2013;18(3):206–14. Epub 2012/12/18. doi: 10.1111/hel.12031 . 23241101 10.1111/hel.12031

[6] Khademi F, Poursina F, Hosseini E, Akbari M, Safaei HG. Helicobacter pylori in Iran: A systematic review on the antibiotic resistance. Iranian journal of basic medical sciences. 2015;18(1):2–7. Epub 2015/03/27. . 25810869 PMC4366738

[7] Karamanolis GP, Daikos GL, Xouris D, Goukos D, Delladetsima I, Ladas SD. The evolution of Helicobacter pylori antibiotics resistance over 10 years in Greece. Digestion. 2014;90(4):229–31. Epub 2014/12/23. doi: 10.1159/000369898 . 25531953 10.1159/000369898

[8] Megraud F, Coenen S, Versporten A, Kist M, Lopez-Brea M, Hirschl AM, et al Helicobacter pylori resistance to antibiotics in Europe and its relationship to antibiotic consumption. Gut. 2013;62(1):34–42. Epub 2012/05/15. doi: 10.1136/gutjnl-2012-302254 . 22580412 10.1136/gutjnl-2012-302254

[9] Boyanova L, Gergova G, Evstatiev I, Spassova Z, Kandilarov N, Yaneva P, et al Helicobacter pylori resistance to six antibiotics by two breakpoint systems and resistance evolution in Bulgaria. Infectious diseases (London, England). 2016;48(1):56–62. Epub 2015/10/16. doi: 10.3109/23744235.2015.1082035 . 26465202 10.3109/23744235.2015.1082035

[10] Shiota S, Reddy R, Alsarraj A, El-Serag HB, Graham DY. Antibiotic Resistance of Helicobacter pylori Among Male United States Veterans. Clinical gastroenterology and hepatology: the official clinical practice journal of the American Gastroenterological Association. 2015;13(9):1616–24. Epub 2015/02/15. doi: 10.1016/j.cgh.2015.02.005 . 25681693 10.1016/j.cgh.2015.02.005 PMC6905083

[11] Liu WZ, Xie Y, Cheng H, Lu NH, Hu FL, Zhang WD, et al Fourth Chinese National Consensus Report on the management of Helicobacter pylori infection. Journal of digestive diseases. 2013;14(5):211–21. Epub 2013/01/11. doi: 10.1111/1751-2980.12034 . 23302262 10.1111/1751-2980.12034

[12] Yuan Y, Ford AC, Khan KJ, Gisbert JP, Forman D, Leontiadis GI, et al Optimum duration of regimens for Helicobacter pylori eradication. The Cochrane database of systematic reviews. 2013;(12):Cd008337 Epub 2013/12/18. doi: 10.1002/14651858.CD008337.pub2 . 24338763 10.1002/14651858.CD008337.pub2 PMC11841770

[13] Liou JM, Chen CC, Lee YC, Chang CY, Wu JY, Bair MJ, et al Systematic review with meta-analysis: 10- or 14-day sequential therapy vs. 14-day triple therapy in the first line treatment of Helicobacter pylori infection. Alimentary pharmacology & therapeutics. 2016;43(4):470–81. Epub 2015/12/17. doi: 10.1111/apt.13495 . 26669729 10.1111/apt.13495

[14] Villoria A, Garcia P, Calvet X, Gisbert JP, Vergara M. Meta-analysis: high-dose proton pump inhibitors vs. standard dose in triple therapy for Helicobacter pylori eradication. Alimentary pharmacology & therapeutics. 2008;28(7):868–77. Epub 2008/07/23. doi: 10.1111/j.1365-2036.2008.03807.x . 18644011 10.1111/j.1365-2036.2008.03807.x

[15] Xin Y, Manson J, Govan L, Harbour R, Bennison J, Watson E, et al Pharmacological regimens for eradication of Helicobacter pylori: an overview of systematic reviews and network meta-analysis. BMC gastroenterology. 2016;16(1):80 Epub 2016/07/28. doi: 10.1186/s12876-016-0491-7 . 27460211 10.1186/s12876-016-0491-7 PMC4962503

[16] Chen H, Dang Y, Zhou X, Liu B, Liu S, Zhang G. Tailored Therapy Versus Empiric Chosen Treatment for Helicobacter pylori Eradication: A Meta-Analysis. Medicine. 2016;95(7):e2750 Epub 2016/02/18. doi: 10.1097/MD.0000000000002750 . 26886617 10.1097/MD.0000000000002750 PMC4998617

[17] Luther J, Higgins PD, Schoenfeld PS, Moayyedi P, Vakil N, Chey WD. Empiric quadruple vs. triple therapy for primary treatment of Helicobacter pylori infection: Systematic review and meta-analysis of efficacy and tolerability. The American journal of gastroenterology. 2010;105(1):65–73. Epub 2009/09/17. doi: 10.1038/ajg.2009.508 . 19755966 10.1038/ajg.2009.508

[18] Marin AC, McNicholl AG, Gisbert JP. A review of rescue regimens after clarithromycin-containing triple therapy failure (for Helicobacter pylori eradication). Expert opinion on pharmacotherapy. 2013;14(7):843–61. Epub 2013/03/30. doi: 10.1517/14656566.2013.782286 . 23537368 10.1517/14656566.2013.782286

[19] Roghani HS, Massarrat S, Pahlewanzadeh MR, Dashti M. Effect of two different doses of metronidazole and tetracycline in bismuth triple therapy on eradication of Helicobacter pylori and its resistant strains. European journal of gastroenterology & hepatology. 1999;11(7):709–12. Epub 1999/08/13. . 10445787 10.1097/00042737-199907000-00004

[20] Fattahi E, Motamedi R, Nayebi AR, Rezazadeh H, Shakir A. Triple therapy using two dosages of metronidazole along with amoxicillin and omeprazole to eradicate Helicobacter pylori infection: a randomized, open study. Indian journal of gastroenterology: official journal of the Indian Society of Gastroenterology. 2004;23(4):154 Epub 2004/08/31. . 15333981

[21] Bardhan K, Bayerdorffer E, Veldhuyzen Van Zanten SJ, Lind T, Megraud F, Delchier JC, et al The HOMER Study: the effect of increasing the dose of metronidazole when given with omeprazole and amoxicillin to cure Helicobacter pylori infection. Helicobacter. 2000;5(4):196–201. Epub 2001/02/17. . 11179983 10.1046/j.1523-5378.2000.00030.x

[22] Matsuhisa T, Kawai T, Masaoka T, Suzuki H, Ito M, Kawamura Y, et al Efficacy of metronidazole as second-line drug for the treatment of Helicobacter pylori Infection in the Japanese population: a multicenter study in the Tokyo Metropolitan Area. Helicobacter. 2006;11(3):152–8. Epub 2006/05/11. doi: 10.1111/j.1523-5378.2006.00394.x . 16684262 10.1111/j.1523-5378.2006.00394.x

[23] Neil GA, Suchower LJ, Ronca PD, Skoglund ML. Time of Helicobacter pylori eradication assessment following treatment. Helicobacter. 1997;2(1):13–20. Epub 1997/03/01. . 9432316 10.1111/j.1523-5378.1997.tb00051.x

[24] Srivastava A. Endoscopic treatment versus endoscopic plus pharmacologic treatment for acute variceal bleeding: a meta-analysis. Indian journal of gastroenterology: official journal of the Indian Society of Gastroenterology. 2002;21(4):169 Epub 2002/10/19. . 12385561

[25] Higgins JP, Thompson SG, Deeks JJ, Altman DG. Measuring inconsistency in meta-analyses. BMJ (Clinical research ed). 2003;327(7414):557–60. Epub 2003/09/06. doi: 10.1136/bmj.327.7414.557 ; PubMed Central PMCID: PMCPmc192859. 12958120 10.1136/bmj.327.7414.557 PMC192859

[26] Lahaie R, Farley A, Dallaire C, Archambault A, Fallone CA, Ponich T, et al Bismuth-based quadruple therapy with bismuth subcitrate, metronidazole, tetracycline and omeprazole in the eradication of Helicobacter pylori. Canadian journal of gastroenterology = Journal canadien de gastroenterologie. 2001;15(9):581–5. Epub 2001/09/27. . 11573100 10.1155/2001/305756

[27] Sierra F, Forero JD, Rey M, Botero ML, Cardenas A. Pilot study: miscellaneous therapy is highly successful for Helicobacter pylori eradication. Alimentary pharmacology & therapeutics. 2013;37(12):1165–71. Epub 2013/05/10. doi: 10.1111/apt.12329 . 23656465 10.1111/apt.12329

[28] Sanchez-Delgado J, Garcia-Iglesias P, Castro-Fernandez M, Bory F, Barenys M, Bujanda L, et al High-dose, ten-day esomeprazole, amoxicillin and metronidazole triple therapy achieves high Helicobacter pylori eradication rates. Alimentary pharmacology & therapeutics. 2012;36(2):190–6. Epub 2012/05/18. doi: 10.1111/j.1365-2036.2012.05137.x . 22591220 10.1111/j.1365-2036.2012.05137.x

[29] Dzieniszewski J, Jarosz M. Guidelines in the medical treatment of Helicobacter pylori infection. Journal of physiology and pharmacology: an official journal of the Polish Physiological Society. 2006;57 Suppl 3:143–54. Epub 2006/10/13. . 17033112

[30] Shimoyama T, Fukuda S, Mikami T, Fukushi M, Munakata A. Efficacy of metronidazole for the treatment of clarithromycin-resistant Helicobacter pylori infection in a Japanese population. Journal of gastroenterology. 2004;39(10):927–30. Epub 2004/11/19. doi: 10.1007/s00535-004-1424-8 . 15549444 10.1007/s00535-004-1424-8

[31] Dore MP, Marras L, Maragkoudakis E, Nieddu S, Manca A, Graham DY, et al Salvage therapy after two or more prior Helicobacter pylori treatment failures: the super salvage regimen. Helicobacter. 2003;8(4):307–9. Epub 2003/09/03. . 12950603 10.1046/j.1523-5378.2003.00150.x

[32] Fraser AG, Moore L, Ali MR, Chua LE, Hollis B, Little SV. An audit of low dose triple therapy for eradication of Helicobacter pylori. The New Zealand medical journal. 1996;109(1027):290–2. Epub 1996/08/09. . 8773671

[33] Bayerdorffer E, Lonovics J, Dite P, Diete U, Domjan L, Kisfalvi I, et al Efficacy of two different dosage regimens of omeprazole, amoxycillin and metronidazole for the cure of Helicobacter pylori infection. Alimentary pharmacology & therapeutics. 1999;13(12):1639–45. Epub 1999/12/14. . 10594399 10.1046/j.1365-2036.1999.00606.x

[34] Suarez MS, Gonzalez Cansino J, Velasco Ilizalde C, Sabatier CA, Castillo Hernandez J. Three treatment schemes with colloidal bismuth subcitrate (Q-ULCER) in peptic ulcer with Helicobacter pylori. Archives of medical research. 1999;30(1):55–9. Epub 1999/03/11. . 10071426 10.1016/s0188-0128(98)00009-8

[35] Heo J, Jeon SW, Jung JT, Kwon JG, Kim EY, Lee DW, et al A randomised clinical trial of 10-day concomitant therapy and standard triple therapy for Helicobacter pylori eradication. Digestive and liver disease: official journal of the Italian Society of Gastroenterology and the Italian Association for the Study of the Liver. 2014;46(11):980–4. Epub 2014/08/19. doi: 10.1016/j.dld.2014.07.018 . 25132282 10.1016/j.dld.2014.07.018

[36] Matougui N, Boudjella ML, Mouffok F, Bouhadef A, Guechi Z, Bouzid K, et al., editors. H. pylori Eradication by Four Triple Therapies: Randomized Study in Double-Blind. International Workshop on Helicobacter and Related Bacteria in; 2009.

[37] Miehlke S, Kirsch C, Schneider-Brachert W, Haferland C, Neumeyer M, Bastlein E, et al A prospective, randomized study of quadruple therapy and high-dose dual therapy for treatment of Helicobacter pylori resistant to both metronidazole and clarithromycin. Helicobacter. 2003;8(4):310–9. Epub 2003/09/03. . 12950604 10.1046/j.1523-5378.2003.00158.x

[38] Nambu K, Tamura A, Miyasaka N, Sugimoto H. THE LOW DOSE ANTIBACTERIAL TREATMENT OF HELICOBACTER PYLORI (HP) INFECTION FOR PEPTIC ULCER. Journal of the Showa University Society. 2000;60.

[39] Sharara AI, Sarkis FS, El-Halabi MM, Malli A, Mansour NM, Azar C, et al Challenging the dogma: a randomized trial of standard vs. half-dose concomitant nonbismuth quadruple therapy for Helicobacter pylori infection. United European gastroenterology journal. 2014;2(3):179–88. Epub 2014/11/02. doi: 10.1177/2050640614530919 . 25360301 10.1177/2050640614530919 PMC4212457

[40] Borody TJ, Brandl S, Andrews P, Ferch N, Jankiewicz E, Hyland L. Use of high efficacy, lower dose triple therapy to reduce side effects of eradicating Helicobacter pylori. The American journal of gastroenterology. 1994;89(1):33–8. Epub 1994/01/01. . 8273794

[41] Nishizawa T, Maekawa T, Watanabe N, Harada N, Hosoda Y, Yoshinaga M, et al Clarithromycin Versus Metronidazole as First-line Helicobacter pylori Eradication: A Multicenter, Prospective, Randomized Controlled Study in Japan. Journal of clinical gastroenterology. 2015;49(6):468–71. Epub 2014/06/13. doi: 10.1097/MCG.0000000000000165 . 24921211 10.1097/MCG.0000000000000165

[42] Di Giulio M, Di Campli E, Di Bartolomeo S, Cataldi V, Marzio L, Grossi L, et al In vitro antimicrobial susceptibility of Helicobacter pylori to nine antibiotics currently used in Central Italy. Scandinavian journal of gastroenterology. 2016;51(3):263–9. Epub 2015/11/12. doi: 10.3109/00365521.2015.1092577 . 26554617 10.3109/00365521.2015.1092577

[43] Kalsch J, Keskin H, Schutte A, Baars T, Baba HA, Bechmann LP, et al Patients with ultrasound diagnosis of hepatic steatosis are at high metabolic risk. Zeitschrift fur Gastroenterologie. 2016;54(12):1312–9. Epub 2016/12/10. doi: 10.1055/s-0042-121899 . 27936481 10.1055/s-0042-121899

[44] Gisbert JP, Calvet X, Gomollon F, Mones J. [Eradication treatment of Helicobacter pylori. Recommendations of the II Spanish Consensus Conference]. Medicina clinica. 2005;125(8):301–16. Epub 2005/09/15. . 16159556 10.1157/13078424

[45] Fock KM, Katelaris P, Sugano K, Ang TL, Hunt R, Talley NJ, et al Second Asia-Pacific Consensus Guidelines for Helicobacter pylori infection. Journal of gastroenterology and hepatology. 2009;24(10):1587–600. Epub 2009/10/01. doi: 10.1111/j.1440-1746.2009.05982.x . 19788600 10.1111/j.1440-1746.2009.05982.x

[46] Su P, Li Y, Li H, Zhang J, Lin L, Wang Q, et al Antibiotic resistance of Helicobacter pylori isolated in the Southeast Coastal Region of China. Helicobacter. 2013;18(4):274–9. Epub 2013/02/20. doi: 10.1111/hel.12046 . 23418857 10.1111/hel.12046

[47] Graham DY. Antibiotic resistance in Helicobacter pylori: implications for therapy. Gastroenterology. 1998;115(5):1272–7. Epub 1998/10/31. . 9797384 10.1016/s0016-5085(98)70100-3

[48] Street ME, Caruana P, Caffarelli C, Magliani W, Manfredi M, Fornaroli F, et al Antibiotic resistance and antibiotic sensitivity based treatment in Helicobacter pylori infection: advantages and outcome. Archives of disease in childhood. 2001;84(5):419–22. Epub 2001/04/24. doi: 10.1136/adc.84.5.419 . 11316688 10.1136/adc.84.5.419 PMC1718745

[49] Bontems P, Kalach N, Oderda G, Salame A, Muyshont L, Miendje DY, et al Sequential therapy versus tailored triple therapies for Helicobacter pylori infection in children. Journal of pediatric gastroenterology and nutrition. 2011;53(6):646–50. Epub 2011/06/28. doi: 10.1097/MPG.0b013e318229c769 . 21701406 10.1097/MPG.0b013e318229c769

[50] Bang CS, Baik GH. Attempts to enhance the eradication rate of Helicobacter pylori infection. World journal of gastroenterology. 2014;20(18):5252–62. Epub 2014/05/17. doi: 10.3748/wjg.v20.i18.5252 . 24833855 10.3748/wjg.v20.i18.5252 PMC4017040

