# Supplementary material for: Meta-analysis: High-dose vs. low-dose metronidazole-containing therapies for Helicobacter pylori eradication treatment
Source: PLoS One. 2018 Jan 25;13(1):e0189888. doi: 10.1371/journal.pone.0189888 (PMC5784897; doi:10.1371/journal.pone.0189888)
Supplement: S1 File — (DOCX) [file pone.0189888.s001.docx]

**Figure legends:**

**Fig 1: Flow chart of literature review.**

**Fig 2: Forest plot of H. pylori eradication rate (intention-to-treat, ITT) with high-dose therapies compared to low-dose therapies.**

**Fig 3: Forest plot of H. pylori eradication rate (per-protocol, PP) with high-dose therapies compared to low-dose therapies.**

**Fig 4: Forest plot of H. pylori eradication rate with high-dose therapies compared to low-dose therapies in the areas with high vs. low metronidazole resistance.** A. Comparison of the high-dose therapies with low-dose therapies in low metronidazole-resistant areas. B. Comparison of the high-dose therapies with low-dose therapies in high metronidazole-resistant areas.

**Fig 5: Forest plot of H. pylori eradication rate with high-dose therapies compared to low-dose therapies in the metronidazole-resistant vs. susceptible people.** A. Comparison of the high-dose therapies with low-dose therapies in metronidazole-resistant people. B. Comparison of the high-dose therapies with low-dose therapies in metronidazole-susceptible people.

**Fig 6: Forest plot of compliance difference between high-dose therapies and low-dose therapies.**

**Fig 7: Forest plot of high-dose therapies vs. low dose-dose therapies in total side effects.**

**S1 Fig: Funnel plot assessing publication bias**

**S2 Fig: Forest plot of high-dose therapies vs. low-dose therapies in particular adherence effects.**
