# Supplementary material for: Meta-analysis: High-dose vs. low-dose metronidazole-containing therapies for Helicobacter pylori eradication treatment
Source: PLoS One. 2018 Jan 25;13(1):e0189888. doi: 10.1371/journal.pone.0189888 (PMC5784897; doi:10.1371/journal.pone.0189888)
Supplement: S2 File — Search strategies for Pubmed, EMBASE and The Cochrane Library database. (DOCX) [file pone.0189888.s002.docx]

**Appendix 1:** **Search strategies for Pubmed, EMBASE and The Cochrane Library database**

**Pubmed**

1. Helicobacter pylori[Mesh]

2. Campylobacter pylori[Title/Abstract]

3.1 or 2

4. metronidazole[Mesh]

5. 2-Methyl-5-nitroimidazole-1-ethanol[Title/Abstract]

6. 2 Methyl 5 nitroimidazole 1 ethanol[Title/Abstract]

7. Trichazol[Title/Abstract]

8. Trichopol [Title/Abstract]

9. Trivazol [Title/Abstract]

10. Vagilen[Title/Abstract]

11. Bayer 5360[Title/Abstract]

12. Clont[Title/Abstract]

13. Danizol[Title/Abstract]

14. Flagyl[Title/Abstract]

15. Gineflavir[Title/Abstract]

16. Metric[Title/Abstract]

17. Metrodzhil[Title/Abstract]

18. MetroGel[Title/Abstract]

19. Metrogyl [Title/Abstract]

20. Metronidazole Hydrochloride[Title/Abstract]

21. Hydrochloride, Metronidazole[Title/Abstract]

22. Metronidazole Monohydrochloride[Title/Abstract]

23. Monohydrochloride, Metronidazole [Title/Abstract]

24. Metronidazole Phosphate[Title/Abstract]

25. Phosphate, Metronidazole[Title/Abstract]

26. Metronidazole Phosphoester[Title/Abstract]

27. Phosphoester, Metronidazole[Title/Abstract]

28. Satric[Title/Abstract]

29. 4 or 5 or 6 or 7 or 8 or 9 or 10 or 11 or 12 or 13 or 14 or 15 or 16 or 17 or 18 or 19 or 20 or 21 or 22 or 23 or 24 or 25 or 26 or 27 or 28

30. Therapeutics[Title/Abstract]

31. Therapeutic[Title/Abstract]

32. Therapy[Title/Abstract]

33. Therapies[Title/Abstract]

34. Treatment[Title/Abstract]

35. Treatments[Title/Abstract]

36. 30 or 31 or 32 or 33 or 34 or 35

37. randomized controlled trial[Publication Type]

38. controlled clinical trial[Publication Type]

39. randomized[Title/Abstract]

40. controlled[Title/Abstract]

41. trial[Title/Abstract]

42. random[Title/Abstract]

43. placebo[Title/Abstract]

44. groups[Title/Abstract]

45. 37 or 38 or 39 or 40 or 41 or 42 or 43 or 44

46. 3 and 29 and 36 and 45

**Embase**

#1 ‘helicobacter pylori’:ab,ti

#2 ‘Campylobacter pylori’:ab,ti

#3 #1 OR #2

#4 ‘metronidazole’:ab,ti

#5 ‘2-Methyl-5-nitroimidazole-1-ethanol’:ab,ti

#6 ‘2 Methyl 5 nitroimidazole 1 ethanol’:ab,ti

#7 ‘Trichazol’:ab,ti

#8 ‘Trichopol’:ab,ti

#9 ‘Trivazol’:ab,ti

#10 ‘Vagilen’:ab,ti

#11 ‘Bayer 5360’:ab,ti

#12 ‘Clont’:ab,ti

#13 ‘Danizol’:ab,ti

#14 ‘Flagyl’:ab,ti

#15 ‘Gineflavir’:ab,ti

#16 ‘Metric’:ab,ti

#17 ‘Metrodzhil’:ab,ti

#18 ‘MetroGel’:ab,ti

#19 ‘Metrogyl’:ab,ti

#20 ‘Metronidazole Hydrochloride’:ab,ti

#21 ‘Hydrochloride, Metronidazole’:ab,ti

#22 ‘Metronidazole Monohydrochloride’:ab,ti

#23 ‘Monohydrochloride, Metronidazole’:ab,ti

#24 ‘Metronidazole Phosphate’:ab,ti

#25 ‘Phosphate, Metronidazole’:ab,ti

#26 ‘Metronidazole Phosphoester’:ab,ti

#27 ‘Phosphoester, Metronidazole’:ab,ti

#28 ‘Satric’:ab,ti

#29 #4 OR #5 OR #6 OR #7 OR #8 OR #9 OR #10 OR #11 OR #12 OR #13 OR #14 OR #15 OR #16 OR #17 OR #18 OR #19 OR #20 OR #21 OR #22 OR #23 OR #24 OR #25 OR #26 OR #27 OR #28

#30 ‘Therapeutics’:ab,ti

#31 ‘Therapeutic’:ab,ti

#32 ‘Therapy’:ab,ti

#33 ‘Therapies’:ab,ti

#34 ‘Treatment’:ab,ti

#35 ‘Treatments’:ab,ti

#36 #30 OR #31 OR #32 OR #33 OR #34 OR #35

#37 ‘randomized controlled trial’

#38 ‘controlled clinical trial’

#39 ‘randomized’

#40 ‘controlled’

#41 ‘trial’

#42 ‘random’

#43 ‘placebo’

#44 ‘groups’

#45 #37 OR #38 OR #39 OR #40 OR #41 OR #42 OR #43 OR #44

#46 #3 AND #29 AND #36 AND #45

**The Cochrane Library**

#1 Campylobacter pylori:ti,ab,kw (Word variations have been searched)

#2 MeSH descriptor: [Helicobacter pylori] explode all trees

#3 #1 OR #2

#4 metronidazole:ti,ab,kw or 2-Methyl-5-nitroimidazole-1-ethanol:ti,ab,kw or 2 Methyl 5 nitroimidazole 1 ethanol:ti,ab,kw or Trichazol:ti,ab,kw or Trichopol:ti,ab,kw or Trivazol:ti,ab,kw or Vagilen:ti,ab,kw or Bayer 5360:ti,ab,kw or Clont:ti,ab,kw or Danizol:ti,ab,kw or Flagyl:ti,ab,kw or Gineflavir:ti,ab,kw or Metric:ti,ab,kw or Metrodzhil:ti,ab,kw or MetroGel:ti,ab,kw or Metrogyl:ti,ab,kw or Metronidazole Hydrochloride:ti,ab,kw or Hydrochloride, Metronidazole:ti,ab,kw or Metronidazole Monohydrochloride:ti,ab,kw or Monohydrochloride, Metronidazole:ti,ab,kw or Metronidazole Phosphate:ti,ab,kw or Phosphate, Metronidazole:ti,ab,kw or Metronidazole Phosphoester:ti,ab,kw or Phosphoester, Metronidazole:ti,ab,kw or Satric(Word variations have been searched)

#5 Therapeutics:ti,ab,kw or Therapeutic:ti,ab,kw or Therapy:ti,ab,kw or Therapies:ti,ab,kw or Treatment:ti,ab,kw or Treatments (Word variations have been searched)

#6 #3 AND #4 AND #5NE.Rep
