# Supplementary figures and images for: Meta-analysis: High-dose vs. low-dose metronidazole-containing therapies for Helicobacter pylori eradication treatment
Source: PLoS One. 2018 Jan 25;13(1):e0189888. doi: 10.1371/journal.pone.0189888 (PMC5784897; doi:10.1371/journal.pone.0189888)

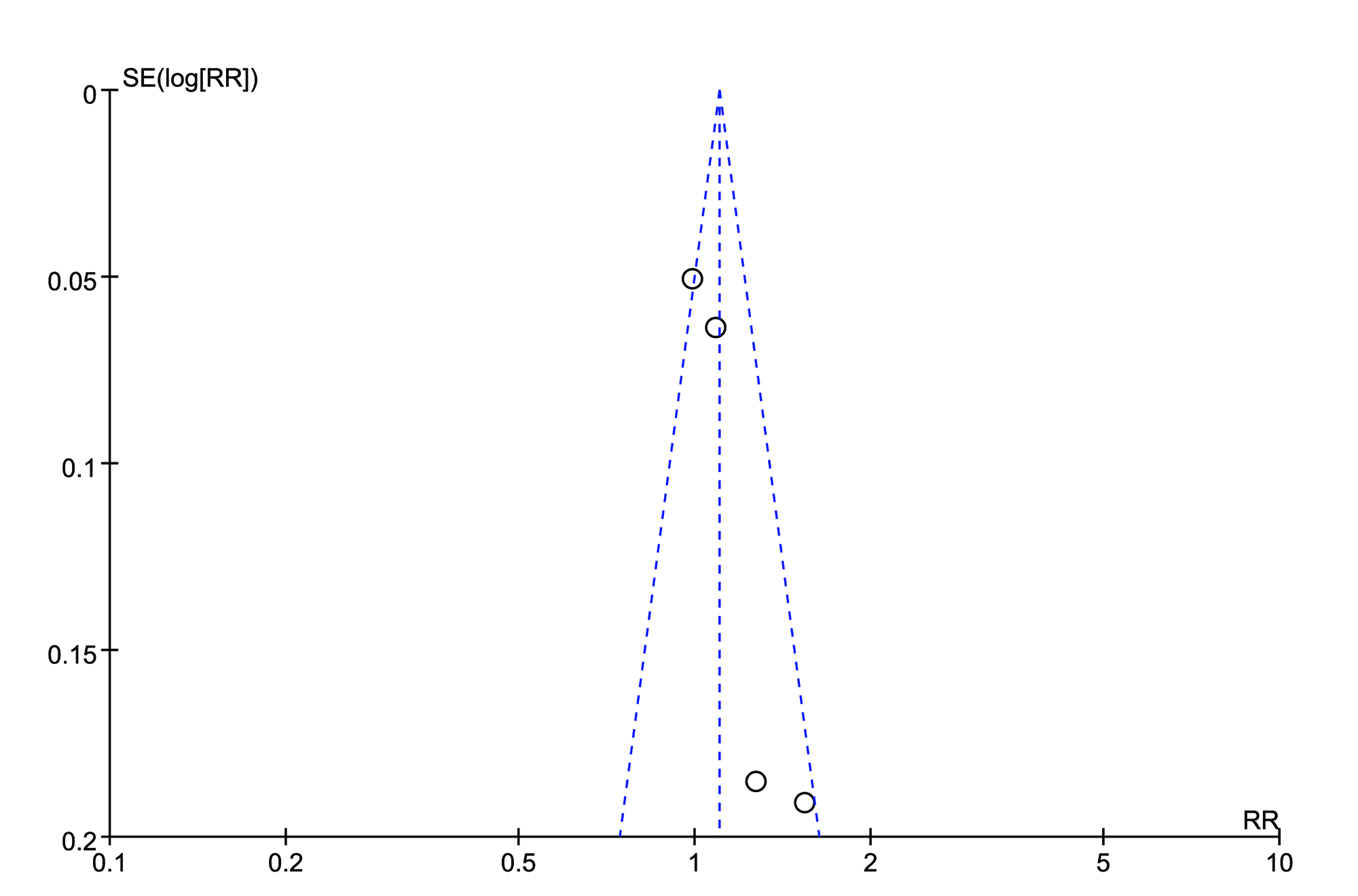

Supplement: S1 Fig — (TIF) [file pone.0189888.s003.tif]

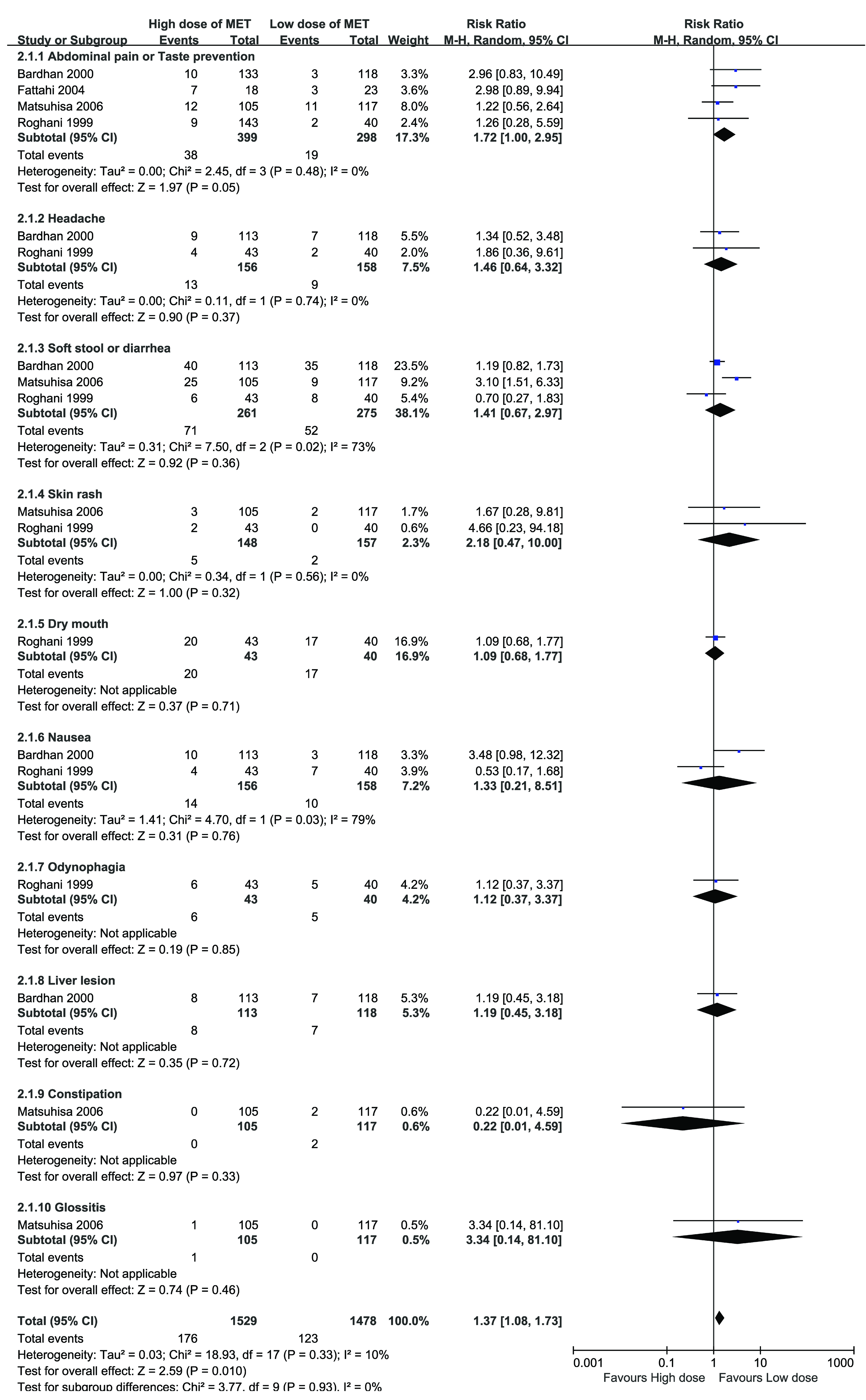

Supplement: S2 Fig — (TIF) [file pone.0189888.s004.tif]
